# A Prospective Study Identifying a Change in Energy and Protein Intake of Older Adults during Inpatient Rehabilitation

**DOI:** 10.3390/nu11020453

**Published:** 2019-02-22

**Authors:** Jorja Collins, Judi Porter, Helen Truby, Catherine E. Huggins

**Affiliations:** 1 Department of Nutrition, Dietetics and Food, Monash University, Notting Hill, VIC 3168, Australia; Judi.porter@monash.edu (J.P.); helen.truby@monash.edu (H.T.); kate.huggins@monash.edu (C.E.H.); 2 Dietetics department, Eastern Health, Box Hill, VIC 3128, Australia; 3 Allied Health Clinical Research Office, Eastern Health, Box Hill, VIC 3128, Australia

**Keywords:** energy intake, protein intake, food consumption patterns, malnutrition, rehabilitation, elderly

## Abstract

Understanding older patients’ dietary intake patterns may improve the timing of strategies to address hospital malnutrition. The aims of this study were to explore longitudinally the change in daily energy (kJ/day) and protein (g/day) intake, and associated factors. Data were derived using a 5-point scale to visually estimate plate waste, and known nutrient composition data. Analyses determined the change in intake between admission and day 14, and admission, day 14 and day 28, respectively. Data were available for 39 participants between admission and day 14 (median (interquartile range) age 82 (78–87) years; 54% male) and 12 participants between admission, day 14 and day 28 (median (IQR) age 79 (69–84) years; 58% male). From admission to day 14 there was a significant increase in the mean (SD) daily intake of energy (6177 (1879) kJ/day vs. 7213 (1903), *p* < 0.001) and protein (63.7 (23) g/day vs. 76.4 (23.0) g/day, *p* = 0.003) but no change from admission to day 14 to day 28. There was a significant inverse relationship between amount consumed at admission and change in intake. Variability in elderly patients’ intake over time has implications for the timing of nutritional care and data collection in research studies.

## 1. Introduction

A number of factors are known to negatively influence food intake of patients in hospital and rehabilitation [1]. Inadequate food intake is common place, with more than half of patients eating a quarter or less of their meal [2]. This contributes to the high prevalence and cost of hospital malnutrition internationally, which particularly affects the elderly [3]. Accurate knowledge of how much patients eat is important for many reasons. Dietary intake data are used by clinicians to determine malnutrition risk, assess nutritional status, and make decisions regarding dietetic referral, review and the provision of nutrition intervention. Dietary intake is also a key outcome in many hospital-based nutrition research studies. Commonly, intake over a short period of time is captured (e.g., 24 h), and little or no consideration is given to the potential influence of the time during admission when data are collected. This may have implications for the clinical decisions and research outcomes relying on this information. 

It is currently unclear how patients’ food intake changes over time during hospitalisation or rehabilitation. Studies prospectively observing and evaluating dietary intake over time provide the strongest evidence to answer this question [4]. Such studies are scarce, and those existing provide limited information [5,6]. Mudge et al. found no significant difference in energy intake between day three and day seven of inpatient stay among 38 general medical patients [5]. Patel et al. assessed plate waste nine times over four weeks and found the proportion of elderly patients eating inadequately (i.e., <75% of meals) was lowest at assessment three and highest at assessment nine, although no statistical analysis was undertaken [6]. 

A number of cross-sectional studies have been undertaken to explore the relationship between dietary intake and length of stay (LOS) [7,8,9,10,11]. As data are collected from each patient once, this study design requires less time and researcher and participant burden. However, findings are unable to reveal how or if intake changes over time for a group of patients. For example, Walton et al. and Kandiah et al. found, respectively, that patients with a longer LOS have lower energy intakes and higher plate waste [7,8]. What these two studies cannot ascertain is whether patients who had a longer LOS had a lower intake during their entire admission or not. Furthermore, the conflicting findings in the body of literature reporting on dietary intake and length of stay adds complexity [7,8,9,10,11].

Longitudinal studies are essential to understand patients’ dietary intake patterns during hospitalisation and rehabilitation. This information will support the development of appropriately timed nutrition screening, assessment and intervention strategies. It could also indicate considerations for dietary intake methods in hospital-based research studies. The aims of these analyses were to explore the longitudinal change in patients’ daily energy and protein intake over two weeks and one month of rehabilitation and identify factors associated with change in intake.

## 2. Materials and Methods 

This study consisted of a secondary analysis of dietary intake data collected prospectively as part of a parallel controlled pilot study evaluating a nutrition intervention registered on the Australian New Zealand Clinical Trials Registry (ANZCTR) (trial ID: ACTRN12613001076763) and reported elsewhere [12]. This study was approved by the Human Research and Ethics Committees of Eastern Health to be completed under a waiver of consent in order to include all eligible patients in order to improve generalisability of primary outcome data.

The study was conducted on a subacute Geriatric Evaluation and Management (GEM) ward in Australia. This ward type is considered slow stream rehabilitation for elderly people with complex, chronic or multiple care needs following an admission to acute hospital [13]. All consecutively admitted patients were recruited unless they met exclusion criteria: having a documented weight loss goal; admitted for palliative care; having complex food requirements; or receiving solely enteral or parenteral nutrition. Participants were allocated to receive the standard menu and usual service of food (control condition) or a higher energy menu and enhanced mid-meal delivery service (intervention condition). Analyses reported here utilise data only from participants allocated to the control condition in order to explore change in intake under usual foodservice and care conditions. Meals were pre-ordered and consumed at the bedside, with food sourced from a cook-chill central production kitchen then heated and plated onsite. A bedside trolley system operated for mid meals. Patients received multidisciplinary care. Referral to a dietitian for assessment and individualised intervention was triggered through a referral and triaging pathway. 

Dietary intake was assessed at three time points: within 72 h of admission, at day 14 and at day 28 of admission for each participant, where length of stay permitted. All data were collected by a single trained research dietitian within ±1 day of designated time points. The primary outcomes of these analyses were change in energy (kJ/day) and protein (g/day) intake between admission and day 14, and between admission and day 28. Foodworks© Version: 7.0 (Xyris Software, Brisbane, Australia) was used to compile the dietary intake information to estimate daily energy and protein intake. The amount of food, drinks and oral nutritional supplements (ONS) consumed on each day of observation was derived from food charts completed by a trained researcher. The quantity of each item remaining on the tray at the end of each meal and mid meal over one day was estimated using a six-point scale (all eaten, one mouthful eaten, ¾, ½, ¼ or none eaten). Visual estimation of each food item by a dietitian has been validated against the weighing method (gold standard) with adequate agreement found [14,15]. Nutrient composition of meals and packaged portion-controlled items were available from the manufacturer [16] and nutrition information panels, respectively. Nutrient information was sourced from AUSNUT database (2007) for miscellaneous items (e.g., vegetables, fruit). Serve sizes were known for portion-controlled items or assumed to be consistent with recommended standards for hospitals [17]. Previous audits established that actual serve sizes were within ±10% of recommended standards. 

Demographic information (age (years), gender and length of stay (LOS)) were collected from medical histories. Nutritional status was assessed at admission by a single trained researcher using the validated Malnutrition Screening Tool (MST) [18] and if MST score ≥2 then a Subjective Global Assessment (SGA) was completed [19]. 

Descriptive statistics were generated for participant characteristics, weight and nutritional status at admission. Paired *t*-test was used to compare mean energy (kJ/day) and protein (g/day) intake at admission and day 14. Repeated measures analysis of variance was used to compare mean energy (kJ/day) and protein (g/day) intake between admission, day 14 and day 28, with Wilk’s Lambda reported to evaluate main effects. Individual participants’ intake between admission and day 14, and admission, day 14 and day 28 are presented graphically to illustrate the direction and magnitude of change. 

Multiple regression was used to explore factors associated with change in intake between admission and day 14. One model was created for protein and another for energy, where the continuous dependent variable was ‘change in intake’ (i.e., day 14 intake − admission intake). Covariates in the model were factors expected to confound intake or potentially explain change (age, nutritional status at admission which was dichotomised as malnourished (code 1) or well nourished (code 0), whether ONS was consumed (yes = code 1, no = code 0), LOS, baseline intake). 

The change in intake between admission and day 14 and factors associated with change in intake between admission and day 14 were repeated after participants staying longer than 14 days were excluded. These sensitivity analyses ascertain the impact on longer-stay patients (who may have poorer health status necessitating longer admission) on the outcomes. Statistical analyses were completed using IBM SPSS (Version 20, Chicago, IL, USA) and *p* < 0.05 was considered statistically significant. 

## 3. Results

### 3.1. Recruitment and Retention 

**Figure 1:**
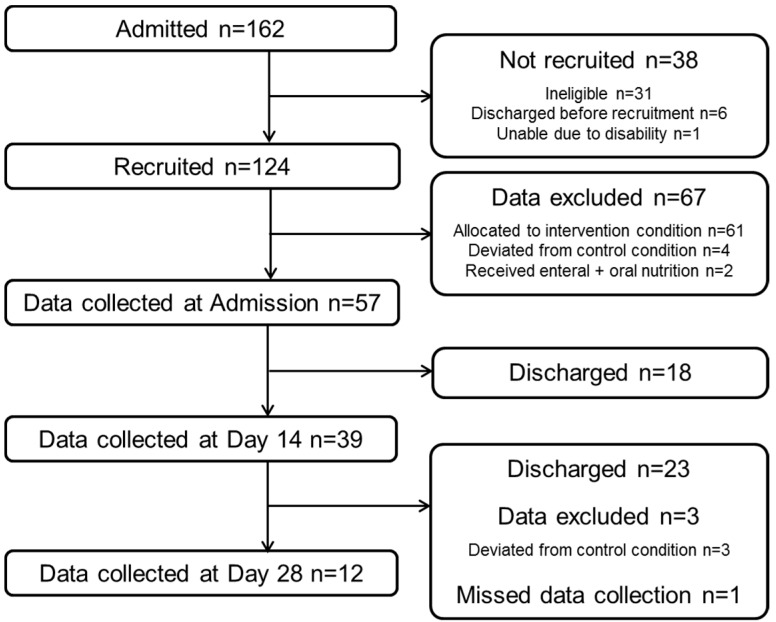
Participant recruitment, retention and data availability.

Of 162 patients admitted during the study period, 124 were recruited. Data were excluded for those who were allocated to the intervention (*n* = 61), deviated from the control condition by changing groups (*n* = 7), missed data collection (*n* = 1) and received enteral and oral nutrition (*n* = 2). Availability of data at each time point was dependent on participants’ length of stay. In total, 72% of participants (41/57) were discharged prior to day 28. Complete data were available to assess change in intake between admission and day 14 for 39 participants, and between admission, day 14 and day 28 for 12 participants. Recruitment, retention and data availability are shown in Figure 1.

### 3.2. Participant Characteristics 

Overall, participants were elderly and the majority were admitted to rehabilitation due to orthopaedic-related diagnoses (Table 1). The prevalence of malnutrition (SGA = B or C) on admission was approximately 40%. Characteristics of participants providing data for analyses of change in intake across both time points are reported in Table 1. There was no difference in characteristics of participants who were and were not included in analyses, with the exception of LOS and weight at admission (Appendix A Table A1 and Table A2). 

### 3.3. Outcomes

Between admission and day 14, there was a significant difference in the average daily intake of energy and protein at the group level (*n* = 39). The mean (SD) intake of energy increased from 6177 (1879) kJ/day at admission to 7213 (1903) kJ/day at day 14 (*p* < 0.001, *n* = 39). Similarly, the mean (SD) protein intake increased from 63.7 (23) g/day at admission to 76.4 (23.0) g/day at day 14 (*p* = 0.003, *n* = 39). These differences remained significant when sensitivity analyses were completed without longer-stay participants (*n* = 27) (data not shown). The number of participants consuming ONS increased from admission (*n* = 12) to day 14 (*n* = 17) while the amount of energy and protein consumed from ONS did not change (mean (SD); energy intake, admission 1923 (827) kJ/day versus day 14 2037 (759) kJ/day, *p* = 0.679; protein intake, admission 20.2 (9.1) g/day versus day 14 22.1 (8.6) g/day, *p* = 0.549; *n* = 11). 

In contrast, between admission, day 14 and day 28, there was no significant change in energy or protein intake among the small group of participants remaining (energy, Wilk’s Lambda = 0.628, *F* (2, 10) = 2.947, *p* = 0.099; protein, Wilk’s Lambda = 0.664, *F* (2, 10) = 2.533, *p* = 0.129; *n* = 12). The mean (SD) intake of energy and protein was 6021 (2392) kJ/day and 58.4 (30.4) g/day at admission, 7048 (2379) kJ/day and 57.3 (24.8) g/day at day 14 and 6431 (2656) kJ/day and 69.4 (32.8) g/day at day 28. 

**Figure 2:**
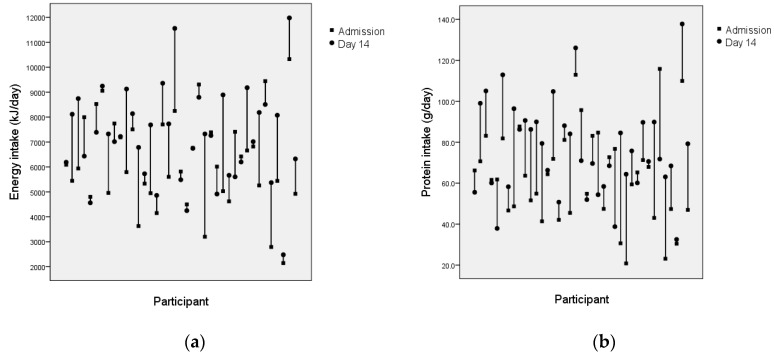
(**a**) Change in energy intake between admission and day 14 for individual participants (*n* = 39). (**b**) Change in protein intake between admission and day 14 for individual participants (*n* = 39).

**Figure 3:**
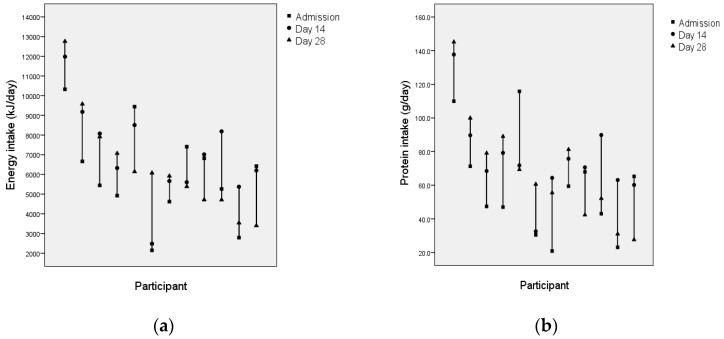
(**a**) Change in energy intake between admission, day 14 and day 28 for individual participants (*n* = 12). (**b**) Change in protein intake between admission, day 14 and day 28 for individual participants (*n* = 12).

At an individual level, change in intake was variable (Figure 2 and Figure 3). Of the 39 participants with data available at admission and day 14, energy intake increased for 24, decreased for 11 and was similar for four. Among the 12 participants with data available for admission, day 14 and day 28, energy intake increased sequentially for five, decreased sequentially for three, and peaked at day 14 for three. Change in protein intake followed a similar pattern to energy.

Change in energy and protein intake from admission to day 14 (Table 2) was significantly associated with amount consumed at baseline; participants consuming the least energy and protein at admission were most likely to have an increase over the first two weeks of rehabilitation. Nutritional status (malnourished or well nourished) was not associated with change in intake. The models performed similarly when sensitivity analyses were completed without longer-stay participants included (*n* = 27) (data not shown). 

## 4. Discussion

This study of elderly patients receiving usual foodservices and multi-disciplinary care during inpatient rehabilitation identified that change in energy and protein intake is highly variable. Among patients with a length of stay of 3.5 weeks (IQR 3–5 weeks), the average energy and protein intake of the group was approximately 1000 kJ and 13 g higher at day 14 than at admission. Two-thirds of patients had an improvement in intake over this two-week period. This occurred among both malnourished and well-nourished patients. Participants with the poorest intake at admission had the greatest improvement in intake, independent of age, LOS, nutritional status and intake of ONS. Conversely, among a smaller subset of participants with a longer length of stay (5 weeks), there was no change overall in energy and protein intake between admission, day 14 and day 28. The absence of change observed over one month may be due to the lack of power as a consequence of the small sample size and the high variability at an individual level.

These prospectively collected data are the first to indicate that a statistically and clinically meaningful (difference of 1000 kJ and 13 g protein between mean intake at admission and discharge) change in intake can occur during rehabilitation. These findings are in line with studies reporting that overall nutritional status is maintained or improved in the majority of elderly patients in subacute care [20,21,22]. The increase in intake observed from admission to day 14 may be due to an improvement in appetite and self-feeding capacity associated with convalescence, and initiation of dietetic intervention. Nevertheless, not all patients had an improvement in intake. Further exploration of the factors contributing to improvement or decline in intake will help to understand if this occurs innately or is a consequence of potentially modifiable processes or time frames of care provision. Our models explained less than a quarter of the variance associated with change in energy or protein intake, so there are other relevant factors at play that were not considered here (e.g., clinical status, habitual intake, socioeconomic status). Further research using a high-quality prospective study design with a large sample size is warranted, particularly to ascertain the dietary intake patterns of longer-stay patients and define who may be at greater risk of nutritional decline. This is a nutritionally vulnerable group with poorer health or function (necessitating longer admission) and higher rates of malnutrition [23,24]. 

The observation that dietary intake may not be consistent at different times during a hospital or rehabilitation stay has implications for clinical practice and hospital-based nutrition research. Strategies to support patients’ intake are most important early on when intake appears to be lowest. Timely malnutrition screening, dietetic referral and triaging pathways are required to minimise the lag time between patient admission and action. This will be achieved through connected, responsive electronic systems for communication and data storage that enable the multidisciplinary team to deliver effective care. For example, electronic medical records that ‘follow’ a patient from an acute hospital into the rehabilitation setting mean relevant information for nutrition screening (e.g., weight history, dietary intake) is on hand, and predefined algorithms can automate a cascade of events if nutrition risk is identified (e.g., dietitian referral). Foodservice systems also have a role to play in delivering high-quality care early on. Menu management systems could generate menus that include high energy and protein options specifically in the first few days of admission. Electronic bedside meal ordering or ‘call centre’ style systems that allow meals to be ordered hours (rather than days) in advance lead to higher energy and protein intake compared to traditional systems [25,26].

Nutrition research studies conducted in healthcare settings need to consider that dietary intake data may be confounded by how long the patient has been in hospital for at the time when data is collected. Steps should be taken in the study design and analysis plan to account for this. Measurement of dietary intake should be undertaken on the same day of admission for all participants (or as close as is feasible). However, this has implications for staffing, as data collectors must be available seven days a week. Alternatively, days since admission should be adjusted for in analyses. This may be preferable for point prevalence studies where data is collected on a single day. 

### Strengths and Limitations

A strength of this study was the prospective design, as it enables the exploration of change within a group over time. However, natural attrition due to discharge from rehabilitation was an associated challenge leading to a modest sample size. This may influence generalisability. It is unclear how dietary intake changes for patients with a LOS of less than 14 days, as data from these individuals were not captured. These findings are unable to be extrapolated to the acute setting due to differences in patient populations and average length of stay. Weighed plate waste is considered the gold standard for determining nutrient intake in a hospital setting, however, the time burden is a challenge and visual observation is used widely as an alternative. The subjectivity of this method is a limitation, although validation studies have shown reasonable agreement with weighed plate waste [14,15]. In this study, all data were collected by a single, experienced observer to eliminate the potential of inter-rater error [27]. 

## 5. Conclusions

These data contribute to the limited existing evidence from prospective studies and suggest that elderly patients’ dietary intake is variable during rehabilitation. Patients with a length of stay of 3.5 weeks consume, on average, more energy and protein at day 14 than they do at admission. This has implications for the provision of foodservice and nutrition care and the timing of dietary assessment, particularly in hospital-based nutrition research.

## Figures and Tables

**Table 1:** Demographic characteristics of study participants included in analyses.

Characteristic		Participants with Data for Admission and Day 14	Participants with Data for Admission, Day 14 and Day 28
		*n* = 39	*n* = 12
Age (years), median (IQR)		82 (78–87)	79 (69–84)
Male, *n* (%)		21 (54)	7 (58)
Impaired cognition, *n* (%)		20 (52)	5 (42)
Diagnosis, *n* (%)	Neurology	1 (3)	
	Orthopaedic/amputation	15 (38)	6 (50)
	Falls/functional decline	6 (15)	2 (17)
	Respiratory/cardiology	6 (15)	
	Cognitive decline	3 (8)	1 (8)
	Gastroenterology/hepatic	3 (8)	2 (17)
	Other	5 (13)	1 (8)
Diet code, *n* (%)	Full ward (unrestricted)	21 (54)	5 (42)
	Soft	2 (5)	1 (8)
	Diabetic	16 (41)	6 (50)
Nutritional status at admission *, *n* (%)	No malnutrition risk	23 (59)	7 (58)
	Mild/moderate malnutrition	12 (31)	3 (25)
	Severe malnutrition	4 (10)	2 (17)
Weight at admission (kg), median (IQR)		71 (56–79)	76 (59–96)
Length of stay, median (IQR)		25 (19–34)	37 (33–52)
FIM score at admission, median (IQR)		72 (57–85)	69 (55–83)

Weight at admission *n* = 2 missing data. * No malnutrition risk, Malnutrition Screening Tool (MST) score > 2 or SGA = A; mild/moderate malnutrition, SGA = B; severe malnutrition, SGA = C. FIM, Functional Independence Measure. IQR, interquartile range.

**Table 2:** Multiple regression models of factors associated with change in energy (kJ/day) and protein (g/day) intake between admission and day 14 of subacute inpatient stay (*n* = 39).

Variable.	B	SEE	Beta	*p* Value
Change in energy intake (kJ/day)				
Age (years)	−2.462	28.722	−0.014	0.932
Length of stay (days)	−13.123	18.998	−0.110	0.495
Nutrition status at admission	433.725	613.002	0.130	0.484
Energy intake at admission (kJ/day)	−0.353	0.131	−0.460	0.011
ONS intake at day 14	707.623	620.103	0.216	0.262
Change in protein intake (g/day)				
Age (years)	−0.357	0.396	−0.135	0.374
Length of stay (days)	−0.036	0.271	−0.020	0.894
Nutrition status at admission	−8.205	8.859	−0.163	0.361
Protein intake at admission (g/day)	−0.608	0.151	−0.633	0.001
ONS intake at day 14	10.050	8.834	0.204	0.264

Model for change in energy intake: SEE = 1512, *R*^2^ = 0.266, Adjusted *R*^2^ = 0.155, overall *p* value = 0.059. Model for change in protein intake: SEE = 21.64, *R*^2^ = 0.337, Adjusted *R*^2^ = 0.237, overall *p* value = 0.015. SEE, standard error of the estimate; B, unstandardised regression coefficient; Beta, standardised regression coefficient; LOS, length of stay; Nutrition status at admission was dichotomised as, malnourished = SGA B or C, well nourished = SGA A or MST < 2; ONS, oral nutritional supplements.

## Appendix A

**Table A1:** Comparison of demographic characteristics of recruited study participants included in analyses and not included in analysis of change in intake between admission and day 14.

Characteristic		Not Included * *n* = 83	Included *n* = 39	*p* Value
Age (years), median (IQR)		83 (74–87)	82 (78–87)	0.982
Male, *n* (%)		40 (48)	21 (54)	0.560
Impaired cognition, n (%)		47 (57)	19 (49)	0.414
Diagnosis, *n* (%)	Neurology	2 (2)	1 (2.6)	
	Orthopaedic/amputation	25 (30)	15 (38.5)	
	Falls/functional decline	17 (21)	6 (15.3)	
	Respiratory/cardiology	13 (16)	6 (15.3)	
	Cognitive decline	1 (1)	3 (7.7)	
	Gastroenterology/hepatic	10 (12)	3 (7.7)	
	Other	15 (18)	5 (12.8)	
Diet code, *n* (%)	Full ward (unrestricted)	56 (68)	21 (54)	0.071
	Soft	9 (11)	2 (5)	
	Diabetic	18 (22)	16 (41)	
Nutritional status at admission **, *n* (%)	No malnutrition risk	52 (63)	23 (59)	0.697
	Malnourished	31 (37)	16 (41)	
Weight at admission (kg), median (IQR)		61.05 (54.45–71.15)	71.15 (56.40–79.20)	0.005
Length of stay, median (IQR)		13 (9–31)	25 (19–34)	<0.001
FIM score at admission, median (IQR)		72 (64–84)	72 (57–85)	0.633

* Not included in analyses due to allocation to the intervention group (*n* = 61), changed group (*n* = 4) or discharged before day 14 (*n* = 18). Data not available for *n* = 2 recruited participants who were excluded from all analyses due to consuming both oral and enteral nutrition. Data missing for: *n* = 5 weight at admission, *n* = 1 FIM score at admission. Data analysed using Chi^2^ test for categorical variables and Mann Whitney U test for continuous variables. ** No malnutrition, MST > 2 or SGA = A; Malnourished, SGA = B or SGA = C. FIM, Functional Independence Measure.

**Table A2:** Comparison of demographic characteristics of recruited study participants included in analyses and not included in analysis of change in intake between admission, day 14 and day 28.

Characteristic		Not included *	Included *n* = 12	*p* Value
		*n* = 110		
Age (years), median (IQR)		83 (75–87)	79 (69–84)	0.167
Male, *n* (%)		54 (49)	7 (58)	0.543
Impaired cognition, *n* (%)		59 (54)	7 (58)	0.757
Diagnosis, *n* (%)	Neurology	3 (3)		
	Orthopaedic/amputation	34 (31)	6 (50)	
	Falls/functional decline	21 (19)	2 (17)	
	Respiratory/cardiology	19 (17)		
	Cognitive decline	3 (3)	1 (8)	
	Gastroenterology/hepatic	11 (10)	2 (17)	
	Other	20 (18)	1 (8)	
Diet code, *n* (%)	Full ward (unrestricted)	72 (66)	5 (42)	
	Soft	10 (9)	1 (8)	
	Diabetic	28 (26)	6 (50)	
Nutritional status at admission **, *n* (%)	No malnutrition risk	68 (62)	7 (58)	
	Malnourished	42 (38)	5 (42)	
Weight at admission (kg), median (IQR)		63.25 (51.50–72.50)	76.10 (59.40–96.20)	0.027
Length of stay, median (IQR)		18 (10–27)	37 (33–52)	<0.001
FIM score at admission, median (IQR)		72 (64–85)	69 (56–83)	0.464

* Not included in analyses due to allocation to the intervention group (*n* = 61), changed group (*n* = 7), discharged before day 28 (*n* = 41) or missed data collection (*n* = 1). Data not available for *n* = 2 recruited participants who were excluded from all analyses due to consuming both oral and enteral nutrition. Data missing for: *n* = 5 weight at admission, *n* = 1 FIM score at admission. Data analysed using Chi^2^ test for categorical variables and Mann Whitney U test for continuous variables. ** No malnutrition, MST > 2 or SGA = A; Malnourished, SGA = B or SGA = C. FIM, Functional Independence Measure.
